# Evaluation of health benefits and harms of the breast cancer screening programme in the Basque Country using discrete event simulation

**DOI:** 10.1186/s12885-015-1700-4

**Published:** 2015-10-12

**Authors:** Arantzazu Arrospide, Montserrat Rue, Nicolien T. van Ravesteyn, Merce Comas, Nerea Larrañaga, Garbiñe Sarriugarte, Javier Mar

**Affiliations:** 1 Gipuzkoa AP-OSI Research Unit, Integrated Health Organization Alto Deba, Avda Navarra 16, 20500 Arrasate-Mondragón, Gipuzkoa Spain; 2 Public Health Division of Gipuzkoa, BIODONOSTIA Research Institute, Paseo Dr Beguiristain s/n, 20014 Donostia, Gipuzkoa Spain; 3 REDISSEC (Red de Investigación en Servicios de Salud en Enfermedades Crónicas – Spanish Health Services Research on Chronic Patients Network), Bilbao, Spain; 4 Basic Medical Sciences department, Biomedical Research Institute of Lleida, University of Lleida, Avda. Rovira Roure 80, 25198 Lleida, Spain; 5 Department of Public Health, Erasmus University Medical Center Rotterdam, Dr Molewaterplein 50, 3015 GE Rotterdam, The Netherlands; 6 Evaluation and Epidemiology Department, Hospital del Mar – IMIM (Hospital del Mar Medical Research Institute), Passeig Maritim 25-29, 08003 Barcelona, Spain; 7 CIBER of Epidemiology and Public Heath, C/Monforte de Lemos 3-5, 28029 Madrid, Spain; 8 Breast Cancer Early Detection Programme, Public Health Division of Bizkaia, Basque Government, Alameda Rekalde 39, 48008 Bilbao, Bizkaia Spain; 9 Health Management Service, Integrated Health Organization Alto Deba, Avda Navarra 16, 20500 Arrasate-Mondragón, Gipuzkoa Spain

**Keywords:** Breast cancer, Evaluation, Public health, Overdiagnosis, Simulation

## Abstract

**Background:**

Since the breast cancer screening programme in the Basque Country (BCSPBC) was started in 1996, more than 400,000 women aged 50 to 69 years have been invited to participate. Based on epidemiological observations and simulation techniques it is possible to extend observed short term data into anticipated long term results. The aim of this study was to assess the effectiveness of the programme through 2011 by quantifying the outcomes in breast cancer mortality, life-years gained, false positive results, and overdiagnosis.

**Methods:**

A discrete event simulation model was constructed to reproduce the natural history of breast cancer (disease-free, pre-clinical, symptomatic, and disease-specific death) and the actual observed characteristics of the screening programme during the evaluated period in the Basque women population. Goodness-of-fit statistics were applied for model validation. The screening effects were measured as differences in benefits and harms between the screened and unscreened populations. Breast cancer mortality reduction and life-years gained were considered as screening benefits, whereas, overdiagnosis and false positive results were assessed as harms. Results for a single cohort were also obtained.

**Results:**

The screening programme yielded a 16 % reduction in breast cancer mortality and a 10 % increase in the incidence of breast cancer through 2011. Almost 2 % of all the women in the programme had a false positive result during the evaluation period. When a single cohort was analysed, the number of deaths decreased by 13 %, and 4 % of screen-detected cancers were overdiagnosed. Each woman with BC detected by the screening programme gained 2.5 life years due to early detection corrected by lead time.

**Conclusions:**

Fifteen years after the screening programme started, this study supports an important decrease in breast cancer mortality due to the screening programme, with reasonable risk of overdiagnosis and false positive results, and sustains the continuation of the breast cancer screening programme in the Basque population.

**Electronic supplementary material:**

The online version of this article (doi:10.1186/s12885-015-1700-4) contains supplementary material, which is available to authorized users.

## Background

Breast cancer (BC), as a disease, meets the criteria necessary for carrying out a population-based screening [1]. The main objective of screening for BC seems as simple as advancing the time of diagnosis and treating the cancer in earlier stages to reduce breast cancer specific mortality. Indeed, overall survival would also increase due to avoided breast cancer. However, although BC screening is beneficial for some, it also may be harmful for others. First, false positive results of mammography can lead to anxiety as they provoke episodes of stress. Second, some women are diagnosed and treated for cancers that, in the absence of screening, never would have become symptomatic (i.e., overdiagnosis) [2].

In most developed countries BC screening is a well-established preventive intervention. Many western European countries implemented mammography screening on the basis of several randomised trials initiated in the 1970s that showed a statistically significant reduction in mortality rates in screened women aged 50 and older [3, 4]. The Health Department of the Basque Government launched the screening programme in 1996, and it was extended to all women aged 50 to 64 years during 1997 and 1998. The decision was held on general recommendations, but there was not informed evidence on its long term impact on the Basque women population with its specific epidemiological characteristics. The BC screening programme in the Basque Country (BCSPBC) has invited more than 400,000 women since 1996. The risk factor profile of the Basque women population would be similar to the screened women population in Catalonia described by Arrospide et al. [5].

Doubts about the balance between screening benefits and harms have increased in recent years [6, 7]. In fact, the Swiss Medical Board published a report in 2013 recommending that systematic mammography screening programmes in which the target population is invited periodically from a population based organisation, should not be introduced and a dead line should be set on existing programmes [8, 9]. The decision regarding continuation of screening should be taken on the basis of the data regularly collected and monitored in local screening programmes, with proper statistical analysis, in addition to the results from the randomized trials [10]. Mathematical models based on the natural history of BC can be helpful to translate those short-term evidence data into long-term health outcomes in order to assess and improve the performance of the programme [11, 12]. Actually, classical methodologies, such as randomised trials, pose major limitations in assessing the long-term effects that are the key outcomes when evaluating health policies such as BC screening. However, those long-term indicators are not considered in the quality indicators proposed by the European Screening Guidelines [13]. Nevertheless, it is possible to extend observed epidemiological short term data into anticipated long term results based on simulation techniques. Several models based on European data have been published [14–17], but very few of those were used to assess the effect of an established screening programme on a real, dynamic population using a multi-cohort model including all the observed cohorts at each time period [18].

The main objective of this study was to assess the health benefit (mortality reduction) and harms (false positive results and overdiagnosis) attributable to the BCSPBC in Basque women since its implementation in 1996 through 2011.

## Methods

A discrete event simulation model [19, 20] was built to reproduce the natural history of BC for women invited to participate in the programme and the characteristics of the BCSPBC since its beginning in 1996 through 2011. The screening programme comprised multiple cohorts of women; a cohort was defined as a group of women invited to participate for the first time in the screening programme in a calendar year. Following the terminology used by Hoyle and Anderson the cohort starting screening in the current year is defined as the incident cohort, and those already undergoing screening from previous years are known as the prevalent cohorts [21]. These terms do not correspond to any disease state in this context but to screening eligibility. Our model reproduced the entire female population invited into the programme during the period 1996–2011. Multiple cohort models, also known as dynamic population models, are used to represent the whole women population that have been invited to the program during the study period whereas single cohort models are useful to conclude lifetime effects in a woman with specific characteristics.

**Fig. 1:**
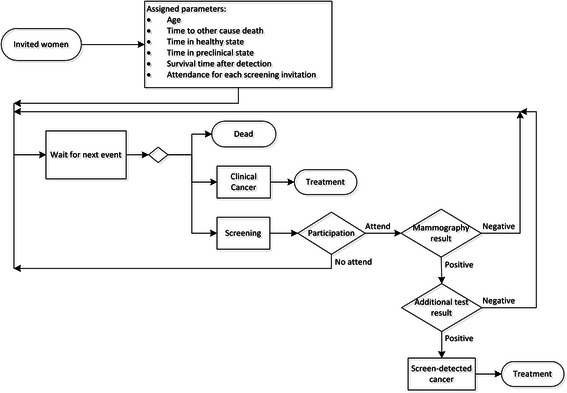
Simplified diagram of the model. Detailed legend: Simplified diagram of the simulation model used to reproduce the natural history of breast cancer for women invited to participate in the programme and the characteristics of the Breast Cancer Screening Programme in the Basque Country

A simplified diagram of the model is shown in Fig. 1 and the data used to implement it is specified in the additional data reported (see Additional file 1: Table S1). The simulation model was constructed using Arena simulation software (Version 14.0, Rockwell software, Milwaukee, WI). No ethical approval or consent was required as no experimental research on humans was involved in this study. However, the Ethics Committee for Clinical Research in Gipuzkoa Health Area evaluated and approved the study.

### Simulated population

The exact number of women invited to participate in the programme for the first time and their ages at that time were available from the programme database. Exactly 414,041 life histories were created, one for every woman invited at least once into the BCSPBC from 1996 through 2011 (see Additional file 1: Table S2).

The age distribution of the invited women changed during the study period. From 1996 to 1998 during the programme implementation, the population consisted only of the incident cohorts aged 50 through 64 years. In subsequent years, instead, the incident cohort included those aged 50 to 51 years. Actually, the target population also included several prevalent cohorts, apart from the incident cohort. The extension of the target population from 50 to 64 years and then 50 to 69 years began in 2006, with women aged 65 years continuing in the programme until age 69.

Mortality from causes other than BC was randomly assigned, depending on the woman’s birth cohort, based on an empirical function. All-cause and BC-caused mortality data were obtained from the Basque mortality registry for the period 1986–2010 (see Additional file 1: Table S1). The high quality of the Basque cancer registry data has been demonstrated by Izarzugaza et al. [22]. The Basque Statistics Institute (EUSTAT) provided the population of Basque women by age and birth cohort. We applied an actuarial method that removes breast cancer as a cause of death to estimate the age at death from causes other than BC, by birth cohort [23].

### Natural history

We modelled the natural history of BC using the approach adopted by Lee et al. [24]. Four main states of health were distinguished: (1) disease-free or undetectable BC; (2) asymptomatic BC that can be diagnosed by screening or preclinical phase; (3) symptomatic BC diagnosed clinically; and (4) death from BC.

The age distribution used to assign the onset of the preclinical phase was obtained from Rue et al. [25]. On the basis of BC incidence from Catalan cancer registries and a distribution of the sojourn time or duration in the pre-clinical state, those authors used a generalized linear model with a Poisson distribution and a polynomial parameterization for the variables of age and cohort for the estimation of BC incidence when no data was available [25]. Cohort effects enabled including upward breast cancer incidence trends in our model. We assumed, as did Lee et al., that the sojourn time of the pre-clinical phase follows an exponential distribution as based on results of clinical trials [24]. The mean value used by Lee et al. for women aged 50 years or more was 4 years.

One of the main assumptions in this model was that every woman who reached the clinical state would be diagnosed clinically at the beginning of this state. Thus, we used the age-specific distribution of BC detection stages observed in the cancer registries of the Basque Country in 1995, before the screening programme began for clinically detected BC (see Additional file 1: Table S3). In situ carcinomas are also included in the model as the lowest stage in which BC could be detected. On the basis of the work by Vilaprinyó et al. [26], we applied distributions of age- and stage-specific survival in women diagnosed either clinically or by screening. Thus, each diagnosed woman was assigned two ages at death and the minimum of these two ages determined the cause and age of death for each woman. Clinical practice and treatments used in detected breast cancers during the studied period followed European guidelines and were described in detail by Arrospide et al. [27].

### Screening effects

The screening test for BCSPBC consisted of mammography with double projection (cranio-caudal and oblique lateral view) carried out biennially [28]. The total number of mammograms performed in the programme was determined by the number of invited women (including early recalls) and annual attendance rates, which were exactly known from the programme data base (see Additonal file 1: Table S2). Annual attendance rates were considered independent as correlation of the participation in first and repeated screening rounds was not available.

Sensitivity and specificity of the entire early detection programme, as well as distribution of breast cancer stage for cancers detected by screening, varied during the study period. Four phases were distinguished: (1) from 1996 to 1999, the implementation phase, when most of the women invited into the programme were incident; (2) from 2000 to 2005, the prevalence phase, when the percentage of women invited for the first time was much lower than the percentage of women invited for successive mammograms; (3) from 2006 to 2008, extension phase, when the programme was extended to women aged 65 to 69 years; (4) from 2009 to 2011, digital phase, when the switch to digital mammography occurred. Sensitivity and specificity of the screening programme for each of the defined phases was calculated on the basis of observed mammography results in the BCSPBC, together with the number of invited women and number of screening-detected breast cancers and observed interval cancers (see Additional file 1: Table S4). In the model, a positive or negative screening result was assigned based on the presence or absence of BC in each woman and the corresponding sensitivity and specificity of the programme.

Distributions of disease stages for screening-detected cases were also obtained for the different phases of BCSPBC (implementation, prevalence, extension and digital) with use of observed data from the screening programme (see Additional file 1: Table S3). This figures were in line with other stage distributions used in similar studies carried out in different countries [17, 24]. In each woman the same random number as the one used for clinical detection stage assignment was used to simulate the stage distribution for screening-detected cancers, in order to estimate the advance in detection stage due to screening.

### Model validation

The model was run in the screened scenario for the whole female population invited at least once into the BCSPBC during the study period in order to reproduce the actual performance of the programme. Model calibration and validation procedure is fully described in the additional file (see Additional file 1). Three main parameters were calibrated: time between consecutive invitations, age distribution of preclinical phase onset and its mean duration. We obtained the best fitting parameters to include in the final model by following the seven-step approach for calibrating models suggested by Karnon et al. [29] and detailed in the additional file (see Additional file 1).

### Screened versus unscreened scenario: outcomes analysis

When the model for the screened scenario was calibrated and validated the same model was run also for the unscreened scenario involving the same female population invited at least once into the BCSPBC during the study period. All the created entities were cloned to obtain two identical populations (screened and unscreened) in each run.

We first used a multi-cohort model that allowed the best approach to reproduce population dynamics in the Basque Country as well as the natural history of breast cancer. However, the assessment of the balance between benefits and harms from preventive programmes typically requires a long follow-up thus the key for its interpretation is to achieve the steady state that is defined as the time when each recently observed behaviour of the system will remain constant in the future [30]. As the first 15 years of the BCSPBC evaluated in this study were not enough to achieve this state, we cannot ensure that the differences between the two scenarios estimated in 2011 will remain in the future.

To further understand the effects of a programme that requires long-term follow-up, we reran the model using a single cohort of 50,000 women aged 50 years who were invited into the programme for the first time in 1996, assuming 100 % participation and life-time follow-up [31]. The inputs used to extrapolate the results from 2011 on were based on the same parameters as those that were used for 2011.

In order to include variability of population characteristics in the model, the multi-cohort model was run 1,000 times. Mean and standard deviations for the results of the 1,000 replications were calculated. The same outputs were obtained for both multi-cohort and single-cohort model. The multi-cohort model was used to estimate population-level effects, whereas individual benefits and harms were estimated with the same model for a single cohort.

The estimated age-specific BC incidence and mortality during the period 1996–2011, which was a scenario that fit the actual development of the screening programme, was compared with the simulated scenario without screening. Overdiagnosis is defined as the number of women who are diagnosed and treated for cases of breast cancer that never would have become symptomatic in the absence of screening. However, the operational definition in the literature used to estimate overdiagnosis is the difference between the number of BC cases detected with screening and the number without screening. In our case as steady state is not achieved, this definition to estimate overdiagnosis includes not only overdiagnosis but also screen detected BC cases that would be clinically detected in the future in absence of screening [32]. Therefore, overdiagnosis is overestimated when analysing population level results in a multi-cohort model that did not achieve the steady state. Accordingly, we will refer in this case to the "incidence increase" instead of "overdiagnosis".

In the case of a single cohort with lifetime follow-up, however the definition used to estimate the incidence increase matches exactly with overdiagnosed cases of BC. We first calculated the relative BC incidence increase (i.e., overdiagnosis) compared with the number of BC cases in a scenario without screening [33] and in a second approach, we estimated the fraction of overdiagnosed cases of BC identified by the screening programme.

In addition, the number of women with a false positive result who were referred to the reference hospital for additional tests based on the sensitivity and specificity data from the BCSPBC were also considered harms of the screening programme.

The probabilities of BC-related death in women detected in the screened and unscreened cohorts were analysed with Cox regression only for the single-cohort analysis. Survival time was measured from the beginning of the assigned clinical phase even for BC detected by screening in order to avoid lead time bias.

## Results

**Fig. 2:**
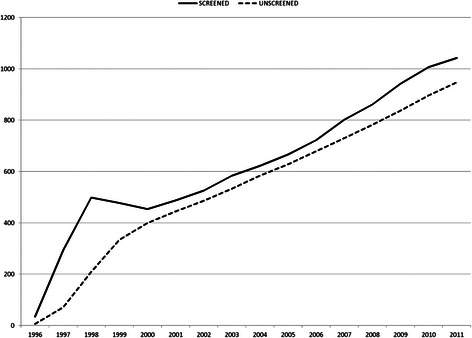
Impact of the screening programme on breast cancer incidence at the population level. Detailed legend: Number of breast cancers detected each year in the invited population estimated using multi-cohort model for the scenarios with and without screening

Table 1 presents the main population-level results (multi-cohort model) of this evaluation in terms of BC incidence and mortality rates per 100,000 women. BC incidence rates for the age groups in the screening programme were calibrated; thus, goodness of fit between the modelled incidence rates and observed rates was acceptable based on the chi-square test. Compared with the scenario without screening, by 2011, the incidence for women aged 50 to 54 years increased by almost 50 % in the scenario with screening, whereas the incidence in age groups between 55 and 69 years increased more than 10 %. For women aged 70 to 74 years, BC incidence decreased by 15 % because of screening in younger ages. The accumulated increase in BC detection attributable to the screening programme from 1996 to 2011 was 17.0 % (Table 2). The extension of the programme to women 69 years of age had a big impact in this figure as, in 2005 alone, before the programme was extended, the increase in diagnosed BC cases was 6.3 %, whereas using data from 2011, the increase in BC incidence rose to 10.2 % (Fig. 2).

**Table 1:** Evolution of estimated breast cancer incidence and mortality rates per 100,000 women in year 2000, 2005 and 2011

		In year 2000		In year 2005		In year 2011	
BC incidence		Mean	95 % CI	Mean	95 % CI	Mean	95 % CI
Screened population							
	<55	316.9	(260.4, 373.4)	285.6	(239.4, 331.8)	310.2	(266.4, 354.0)
	55-59	168.9	(138.9, 198.8)	214.9	(181.1, 248.6)	245.9	(209.9, 281.9)
	60-64	220.5	(180.6, 260.2)	216.9	(182.0, 251.7)	276.1	(237.0, 315.1)
	65-69	162.6	(129.2, 196.0)	199.7	(161.4, 237.8)	264.7	(227.0, 302.3)
	70-74	0	(0.0, 0.0)	201.3	(163.3, 239.2)	251.2	(206.4, 295.9)
	75-79	0	(0.0, 0.0)	0	(0.0, 0.0)	300.2	(253.5, 346.8)
	> = 80	0	(0.0, 0.0)	0	(0.0, 0.0)	94.9	(11.7, 178.1)
Unscreened population							
	<55	180.2	(137.7, 222.7)	188	(150.5, 225.3)	206.7	(171, 242.3)
	55-59	154.5	(125.9, 183.1)	190.8	(159, 222.5)	216.2	(182.4, 249.8)
	60-64	204.7	(166.4, 242.9)	200.1	(166.6, 233.4)	249	(211.9, 285.9)
	65-69	190.6	(154.4, 226.6)	246.3	(203.9, 288.6)	238.4	(202.7, 273.9)
	70-74	0	(0.0, 0.0)	227.2	(186.8, 267.4)	295.9	(247.4, 344.4)
	75-79	0	(0.0, 0.0)	0	(0.0, 0.0)	305.8	(258.6, 352.8)
	> = 80	0	(0.0, 0.0)	0	(0.0, 0.0)	94.9	(11.7, 178.1)
BC mortality							
Screened population							
	<55	5.2	NA	7.8	(0.1, 15.3)	11.3	(2.9, 19.5)
	55-59	8.3	(1.6, 14.9)	27.6	(15.4, 39.6)	35.7	(21.9, 49.4)
	60-64	11.2	(2.2, 20.1)	36.4	(22.1, 50.6)	53.2	(36.0, 70.3)
	65-69	12.5	(3.2, 21.7)	49.4	(30.4, 68.4)	57.1	(39.6, 74.6)
	70-74	0	(0.0, 0.0)	65.2	(43.6, 86.8)	95.5	(67.8, 123.0)
	75-79	0	(0.0, 0.0)	0	(0.0, 0.0)	130.3	(99.5, 161.0)
	> = 80	0	(0.0, 0.0)	0	(0.0, 0.0)	38	NA
Unscreened population							
	<55	10.4	(0.2, 20.6)	13.6	(3.5, 23.6)	17.6	(7.2, 28.0)
	55-59	12.4	(4.3, 20.5)	37.1	(23.0, 51.0)	45.1	(29.7, 60.5)
	60-64	18.6	(7.0, 30.1)	47.8	(31.5, 64.1)	65.8	(46.7, 84.8)
	65-69	19.6	(8.0, 31.1)	64.4	(42.7, 86.0)	74.8	(54.8, 94.7)
	70-74	0	(0.0, 0.0)	74.5	(51.4, 97.5)	115.9	(85.5, 146.2)
	75-79	0	(0.0, 0.0)	0	(0.0, 0.0)	132.1	(101.1, 163.0)
	> = 80	0	(0.0, 0.0)	0	(0.0, 0.0)	38	NA

*BC* Breast cancer, *CI* Confidence interval, *NA* Not applicable

**Table 2:** Accumulated population level results for the period from 1996 to 2011. Detailed legend: Main accumulated results for the period from 1996 to 2011 in the analysis of the multi-cohort model reproducing the population of Basque women invited into the breast cancer screening programme

	Screened population		Unscreened population	
	Mean	95 % CI	Mean	95 % CI
Number of women	411,782	(411,619, 411,945)	411,782	(411,619, 411,945)
Participation rate	77.80 %	(77.6 %, 77.9 %)	0 %	(0.0 %, 0.0 %)
Number of mammograms	1,308,030	(1,304,309, 1,311,750)	0	(0, 0)
False positive results	8,211	(7,876, 8,546)	0	(0, 0)
False positive/women	1.99 %	(1.91 %, 2.08 %)	-	-
False positive/mammography	0.63 %	(0.60 %, 0.65 %)	-	-
Screening-detected BC	5,267	(4,999, 5,535)	0	(0, 0)
Total detected BC	10,021	(9,644, 10,399)	8,567	(8,215, 8,918)
Difference in BC detection	1,454	(1,316, 1,593)	-	-
Increase in BC detection	-	-	17.0 %	(15,2 %, 18,8 %)
BC deaths	1,512	(1,370, 1,655)	1,883	(1,725, 2,041)
Difference in BC deaths	-	-	371	(299, 442)
Difference in BC deaths/BC deaths	-	-	19.7 %	(16.3 %, 23.1 %)

*BC* Breast cancer, *CI* Confidence interval

Among 1,308,030 mammograms performed in the BCSPBC during the evaluated period, 13,478 women with positive mammograms were referred for additional testing at the reference hospital during the study period; 39.1 % of them were diagnosed with BC (Table 2).

**Fig. 3:**
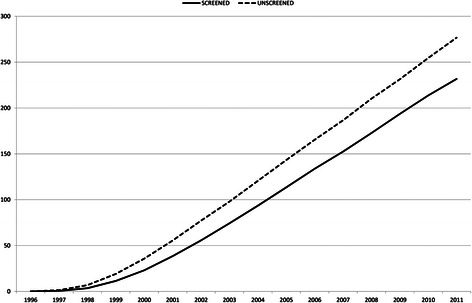
Impact of the screening programme on breast cancer mortality at the population level. Detailed legend: Annual number of deaths due to breast cancer in the invited population estimated using the multi-cohort model for the scenarios with and without screening

Reduction in mortality was greater the more cohorts were included in the programme. During the study period (1996–2011), the screening programme avoided 19.7 % of the deaths due to BC that would have occurred in a scenario without screening (Table 2). Specifically in 2011, the BC mortality rate in women invited into the screening programme was 16.0 % lower than in a scenario without screening (Fig. 3). The screening effect on BC mortality in 2011 was greater for women aged 50 to 55 years, with a 27.3 % decrease than for the rest of the age groups in the screening programme (55–69 years) with a 22.2 % decrease. Women aged 70 or more who previously participated in the programme still had a significant decrease in their probability of death from BC (17.5 % for women aged 70–74 years and 2.8 % for women aged 75–79), compared with the scenario without screening (Table 1).

**Fig. 4:**
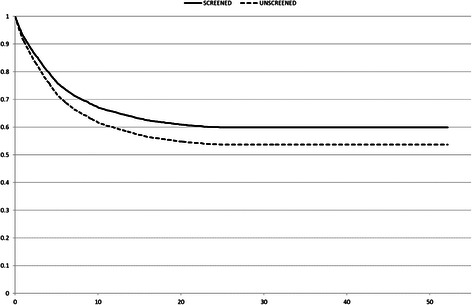
Breast cancer survival analysis corrected by lead time bias. Detailed legend: Breast cancer survival curves corrected by lead time bias among cases detected in women in a single cohort for the scenarios with and without screening

When we analysed the survival time, corrected by lead time, for screening-detected BC cases in a single cohort with lifetime follow-up, the hazard ratio for BC mortality was 0.83 (95 % confidence interval [CI], 0.77-0.89) (Fig. 4). Life-years gained were 32.6 days for each woman invited into the screening programme and 2.5 years gained for each woman with BC detected by the screening programme (Table 3). In this scenario, 1 out of 28 women detected with BC by screening (3.6 %) would be overdiagnosed (Table 3). The mean lead time in BC detection produced by the screening programme, excluding overdiagnosed cases, was 3.2 years (95 % CI, 3.1-3.4).

**Table 3:** Accumulated results for a single cohort of women aged 50 years from 1996 to 2011. Detailed legend: Main results of a single cohort of women aged 50 years invited to participate in the Basque breast cancer screening programme for the first time in 1996

	Screened population		Non-screened population	
	Mean	95 % CI	Mean	95 % CI
Number of women	50,000	(50,000, 50,000)	50,000	(50,000, 50,000)
Screening age	50-69 years old		-	-
Participation rate	100 %		0 %	(0.0 %, 0.0 %)
Number of mammograms	480,869	(480,150, 481,588)	0	(0, 0)
False positive results	3,151	(3,041, 3,260)	0	(0, 0)
False positive/women	6.3 %	(6.1 %, 6.5 %)	-	-
False positive/mammography	0.7 %	(0.6 %, 0.7 %)	-	-
Screen detected BC	1,776	(1,696, 1,856)	0	(0, 0)
Total detected BC	5,065	(4,926, 5,204)	5,001	(4,863, 5,139)
Difference in BC detection (Overdiagnosis)	64	(49, 79)	-	-
Increase in BC incidence	-	-	1.3 %	(1.0 %, 1.6 %)
Overdiagnosis/Screening-detected BC	3.6 %	(2.8 %, 4.4 %)	-	-
BC deaths	1,634	(1,556, 1,711)	1,880	(1,795, 1,964)
Difference in BC deaths	-	-	246	(215, 278)
Difference in BC deaths/BC deaths	-	-	13.1 %	(11.6 %, 14.6 %)
Life years/women	82.6	(82.5, 82.7)	82.5	(82.4, 82.6)
Life years gained/women	0.09	(0.08, 0.10)	-	-
Life years gained/screen detected women	2.5	(2.2, 2.8)	-	-

*BC* Breast cancer, *CI* Confidence interval

## Discussion

Our main finding was that the BCSPBC achieved an important reduction in population level BC mortality in 2011 (16.0 %), with limited adverse effects. Therefore, our results support the continuation of the programme as a public policy aimed at reducing the burden of BC in the Basque population. The estimations for 2011 in terms of BC incidence increase and false positive results were in line with the values described in the literature [2, 33–36]. Planning a population-level breast cancer screening programme can require multiple criteria for decision making; it involves primarily reduction in BC mortality rates, but it also needs to minimize overdiagnosis and false positive events. In our case, as the screening programme was already implemented and the mathematical model was used for evaluation, decision makers' preferences should be considered in the interpretation of the results as no unique optimal solution exists [37].

Evaluating screening health effects is complicated as we are analysing a system in which the natural history of BC and screening effects are combined. To correctly interpret its impact we should ensure that the measured effects will remain steady in the future; otherwise its evaluation could be misleading. The changes in screening features, such as the extension implemented in 2006, modified the evolution of the indicators (i.e. difference in BC incidence) which hamper analysis of the screening programme. Our results highlight that the variability between the results from different studies depends primarily on the characteristics of the screening programme, but it also depends on the actual variation of these features during its implementation. Therefore, we determined the estimated effects using the model for a single cohort as complementary results.

Mortality results are in accordance with the population level results obtained by The Cancer Intervention and Surveillance Modeling Network (CISNET), a consortium of the National Cancer Institute. CISNET results showed a reduction in BC mortality between 7.5 % and 22.7 % in the United States using seven different models [38]. According to the review published by Broeders et al., the mean European estimate of reduction in BC mortality is 25 % to 31 % for invited women [39]. Furthermore, a meta-analysis that included 11 randomized controlled trials with 13 years of follow-up estimated a 20 % reduction in BC mortality (95 % CI 11 %-27 %) [34]. Specifically, in a study carried out in Spain using mathematical models, Carles et al. concluded a 19.6 % reduction in BC mortality, similar to our final estimate [40]. When a single cohort (50 years old women invited for the first time in 1996) was analysed with life-time follow up, we concluded that BC mortality decreased by 13.1 %. Actually, 2.5 years of life were gained for each woman with breast cancer detected by the screening programme.

The Basque population had not arrived at a steady state by 2011, and therefore, a longer follow-up is necessary to estimate overdiagnosis at the population level without overestimating it. When we assessed 10.2 % BC incidence increase in 2011, we included early detected BC cases although they were not necessarily overdiagnoses as they could be detected from 2012 on. A study published by Duffy et al. [41] concluded that a 15 % increase in incidence in the screened group aged 50 to 69 years can persist 30 years after the start of the programme. In concordance, at 29 years follow-up the long-term incidence of breast cancer in the Swedish Two-County Trial of Mammographic screening, Yen et al. conclude that there was no excess of incidence and thus, suggest that overdiagnosis is a minor phenomenon [42].

Actually, when we analysed a single cohort of 50 years old women followed until death, the estimated increase in incidence was 1.3 % of the detected breast cancers compared to a scenario without screening [32]. Gunsoy et al. concluded that 5.6 % of all BC detected were overdiagnosed in a cohort of British women followed up from age 40 to 85 years [15]. The main difference with respect to our 1.3 % of overdiagnosed cases might be due to the mean sojourn time in the model of Gunsoy et al. When Gunsoy et al. used a shorter sojourn time, overdiagnosis decreased to 3.1 % of all BC diagnosed. Furthermore, as in our model, the cohort was followed until death, the number of all BC detected increased, and therefore, the percentage was lower.

Although overdiagnosis could be considered the major harm of screening, false positive mammographic results are also important harms, since they can cause stress and a decrement in quality of life in healthy women. Our single-cohort analysis reported an accumulated risk of 6.3 % for a false positive screening result, with early recall not included as a positive result. False positive rates in our study were concordant with a cumulative chance of 7.3 % estimated in the Nijmegen population-based cohort study [36]. The literature review by Hofvind et al. [35] estimated that the risk of an invasive procedure with benign outcome ranged from 1.8 % to 6.3 % per woman in Europe.

### Strengths

Our study is strengthened by the exhaustive methodology used for the validation process. The robustness of this study is reinforced by the reproduction of the main results obtained during the study period in the observed screened population [43]. Thus, using the model, we were able to estimate the results in a scenario without a screening programme. The detailed mathematical model built to include the progressive dissemination of screening in all the cohorts between 50 and 69 years of age enabled the interpretation of the mortality reduction achieved in the first 15 years of the programme. Complementary single-cohort analysis was also helpful when interpreting population level effects.

### Limitations

One limitation of this study was that it was built exactly for the Basque women population invited to the BCSPBC from 1996 through 2011. However, using mathematical models for the evaluation of an already implemented screening programme is applicable to other populations.

A second limitation was that long-term follow-up made it necessary to make assumptions for the projected years. In this study the detection stage distribution of symptomatic BC from 2011 on was based on the same parameters as in 2011.

Finally, it is worth mentioning that BC survival functions applied in the simulation model relied on age and stage at detection but did not change during the study years. Consequently, the effect of improved treatment in women with BC was not incorporated. This way we ensure the observed effect was of only related to screening.

## Conclusions

In conclusion, this study has assessed the impact of the BCSPBC at the population level in terms of reduction in breast cancer mortality and the number of false positive results and overdiagnosed cases. Fifteen years after the screening programme started, this study supports an important decrease in breast cancer mortality, with reasonable risk of harm with screening and sustains the continuation of the breast cancer screening programme in the Basque population. The analysis of the effects using the same simulation model in a single cohort is complementary to the population-level results and can help in the interpretation of the population-level estimates.

## Additional file

Additional file 1: **Model inputs, calibration and validation.** This file includes model building details related to the simulation model developed for the evaluation of the Breast Cancer Screening Programme in the Basque Country. In particular, programme specific data used as inputs in the model, a detailed description of the model calibration procedure and goodness-of-fit of the final model are shown in this appendix. (PDF 88 kb)

## Abbreviations

BC: Breast cancer
BCSPBC: Breast cancer screening programme in the Basque Country
CISNET: The cancer intervention and surveillance modeling network
EUSTAT: Basque statistics institute
