# Supplementary material for: Evaluation of health benefits and harms of the breast cancer screening programme in the Basque Country using discrete event simulation
Source: BMC Cancer. 2015 Oct 12;15:671. doi: 10.1186/s12885-015-1700-4 (PMC4603694; doi:10.1186/s12885-015-1700-4)

Model inputs, calibration and validation

Three main parameters were calibrated: time between consecutive invitations, age distribution of preclinical phase onset and its mean duration. We obtained the best fitting parameters to include in the final model by following the seven-step approach for calibrating models by Karnon et al. [26].

First, we calibrated the time between intervals considering that it was not influenced by other unobserved parameters. At the beginning, we used a random search algorithm considering different values from a normal distribution centred in 2 years and standard deviation 0.5. Based on these results, we continued using a grid search algorithm, running 25 simulations for 10 different values between 2.11 and 2.20. The goodness-of-fit measure applied to assess the difference between observed and expected outcomes was the chi-square statistic. The overall chi-square statistic of each hypothesis was calculated as the sum of the chi-square statistics calculated for the analysed years. We assumed outcomes for each year were independent. We included in the model the parameter value for which the overall chi-square statistic was the minimum: 2.18 year between consecutive invitations (Figure S1).

Afterwards, we calibrated jointly two factors. The first one will be the relative risk (RR) for the incidence function. The second multiplier will be used to calibrate the mean value for the preclinical state duration which prior estimate was 4.0. Thus we will calibrate the factor t to obtain a final mean preclinical state duration 4t. We considered as target outputs the number of screening-detected cancers from 1996-2011, together with total cancer detection rates by age group (50-54, 55-59, 60-64, 65-69) for the period 1999-2009. Random search algorithm was used also in this case considering Normal(1,0.25) distribution for both factors for a first approximation and a grid search algorithm centred in $0.87 \leq RR \leq 0.90$ and $0.85 \leq t \leq 0.90$. The goodness-of-fit measure used in this case was also the chi-square statistic, and we defined the overall chi-square as the sum of the measures calculated for each target output using the same weight for all five outputs. We included in the model the parameter set for which the overall chi-square statistic was the minimum. The final relative risk used for BC incidence functions was 0.88, and the mean time in preclinical state 3.44 years ($t = 0.86$) (Figure S2).

For model validation, we compared the estimated results for the screened population (multi-cohort model) with the observed indicators from BCSPBC and the Basque cancer registries such as number of

invited women, number of mammograms carried out in the programme, age-specific breast cancer incidence or the number of women with a positive mammography result (Figure S3). We also confirmed that life expectancy for women from the general population and women who died from BC was concordant with the observed data (Table S5).

Table S1: Model input and validation parameters

Input data	Source
Invited population	
Number of women invited for the first time	Screening programme data
Age distribution	Screening programme data
Participation rate	Screening programme data
Time until event	
Other cause mortality	Basque mortality registry
Breast cancer mortality	Basque mortality registry
Time till pre-clinical state	Rue et al, 2009
Pre-clinical state duration	Lee and Zelen, 2006
Age- and stage-specific breast cancer survival	Vilaprinyo et al.
Detection data	
Clinically detected cancer stage distribution	Basque cancer registry
Programme sensitivity and specificity	Screening programme data
Screen detected cancer stage distribution	Screening programme data

Validation data	Source
Invited population	
Total number of invited women	Screening programme data
Total number of mammograms	Screening programme data
Recall rate	Screening programme data
Remitted for additional test	Screening programme data
Detection data	
Age- and year-specific breast cancer incidence	Basque cancer registry
Screening-detected cancers	Screening programme data

Table S2: Number of women invited into the breast cancer screening programme in the Basque Country and participation rates (%).

	First invitations		Successive invitation	
Year	Number of women	Participation	Number of women	Participation
1996	7,835	79.71	0	-
1997	67,719	72.94	0	-
1998	87,967	78.26	16,702	71.49
1999	41,841	84.60	51,037	64.57
2000	17,426	96.27	80,399	74.77
2001	18,902	90.45	86,792	70.82
2002	16,401	90.04	86,110	74.54
2003	21,109	84.38	87,877	74.59
2004	16,363	87.26	86,327	75.08
2005	14,043	89.49	91,996	75.35
2006	16,804	86.39	114,691	73.97
2007	17,018	87.92	105,850	75.18
2008	17,847	83.85	110,542	75.15
2009	18,510	85.68	116,330	75.51
2010	17,711	88.48	120,481	79.45
2011	16,545	91.21	128,836	79.49

Table S3: Distribution of breast cancer detection stages.

Detection stage	In situ	Stage I	Stage IIa	Stage IIb	Stage III	Stage IV
Clinically detected cancer (in 1995)						
50-59	10.00	32.63	24.75	15.75	9.00	7.88
60-69	7.42	21.72	22.86	26.29	13.72	8.00
>69	4.35	12.11	27.85	12.11	24.22	19.37
Screen detected cancer	In situ	Stage I	Stage IIa	Stage IIb	Stage III	Stage IV
Period 1996-1999						
50-59	19.69	49.71	19.30	7.60	3.12	0.58
60-69	17.94	50.93	19.18	6.80	4.12	1.03
Period 2000-2005						
50-59	18.77	49.16	20.34	6.39	4.65	0.70
60-69	18.08	57.18	15.60	5.33	3.43	0.38
Period 2006-2008						
50-59	18.77	49.16	20.34	6.39	4.65	0.70
60-69	18.08	57.18	15.60	5.33	3.43	0.38
Period 2009-2011						
50-59	18.76	49.47	21.18	4.99	4.54	1.06
60-69	15.55	54.50	19.97	5.56	3.93	0.49

Table S4: Sensitivity and specificity of the breast cancer screening programme.

Year	1996-1999	2000-2005	2006-2008	2009-2011
Sensitivity	95.20	83.40	83.52	85.86
Specificity	90.44	90.61	93.67	94.13

Table S5: Validation of the mean life expectancy for women in the general population and median survival time corrected by lead time for women with death from BC.

	Theoretical*	Estimated
General population	83.70	82.61
BC death survival		
Stage I	9.03	6.34
Stage IIa	6.46	4.77
Stage IIb	5.14	4.19
Stage III	3.41	2.74
Stage IV	0.80	0.63

*Median BC survival times when no other cause deaths occur are shown as theoretical. Estimated median survival times for BC deaths are lower than theoretical as women with greater BC survival time die from other causes.

BC = breast cancer

Figure S1: Total number of women invited to join the programme and the number of the mammograms carried out.

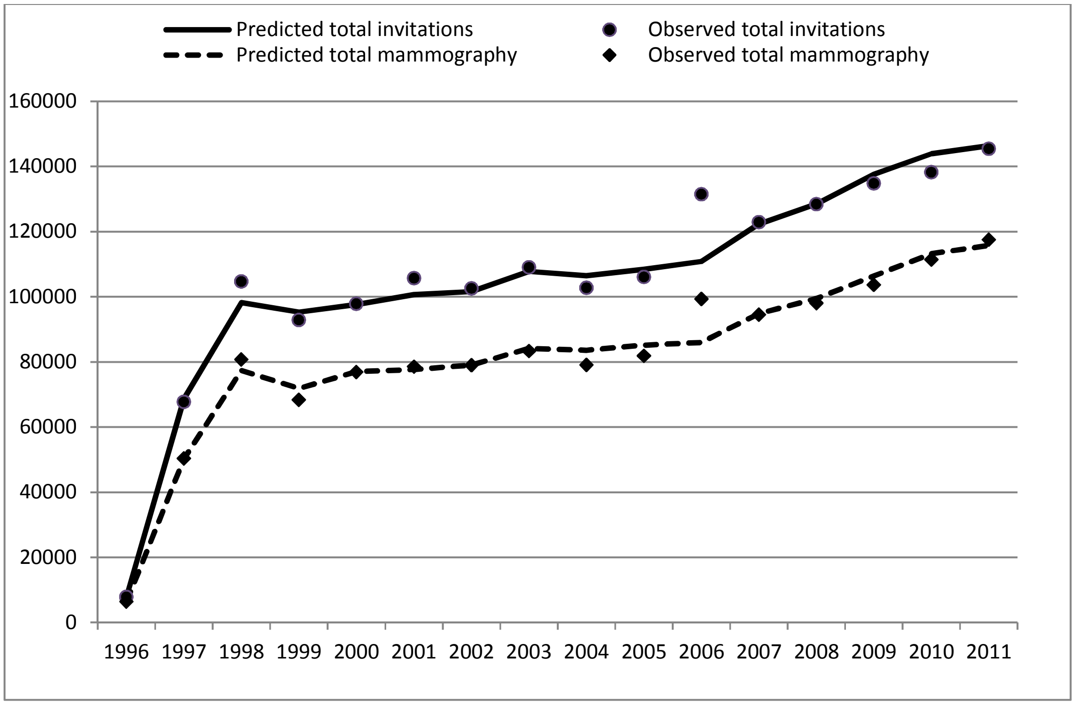

Figure S2: Breast cancer incidence by age group (implementation period 1996-1999 excluded).

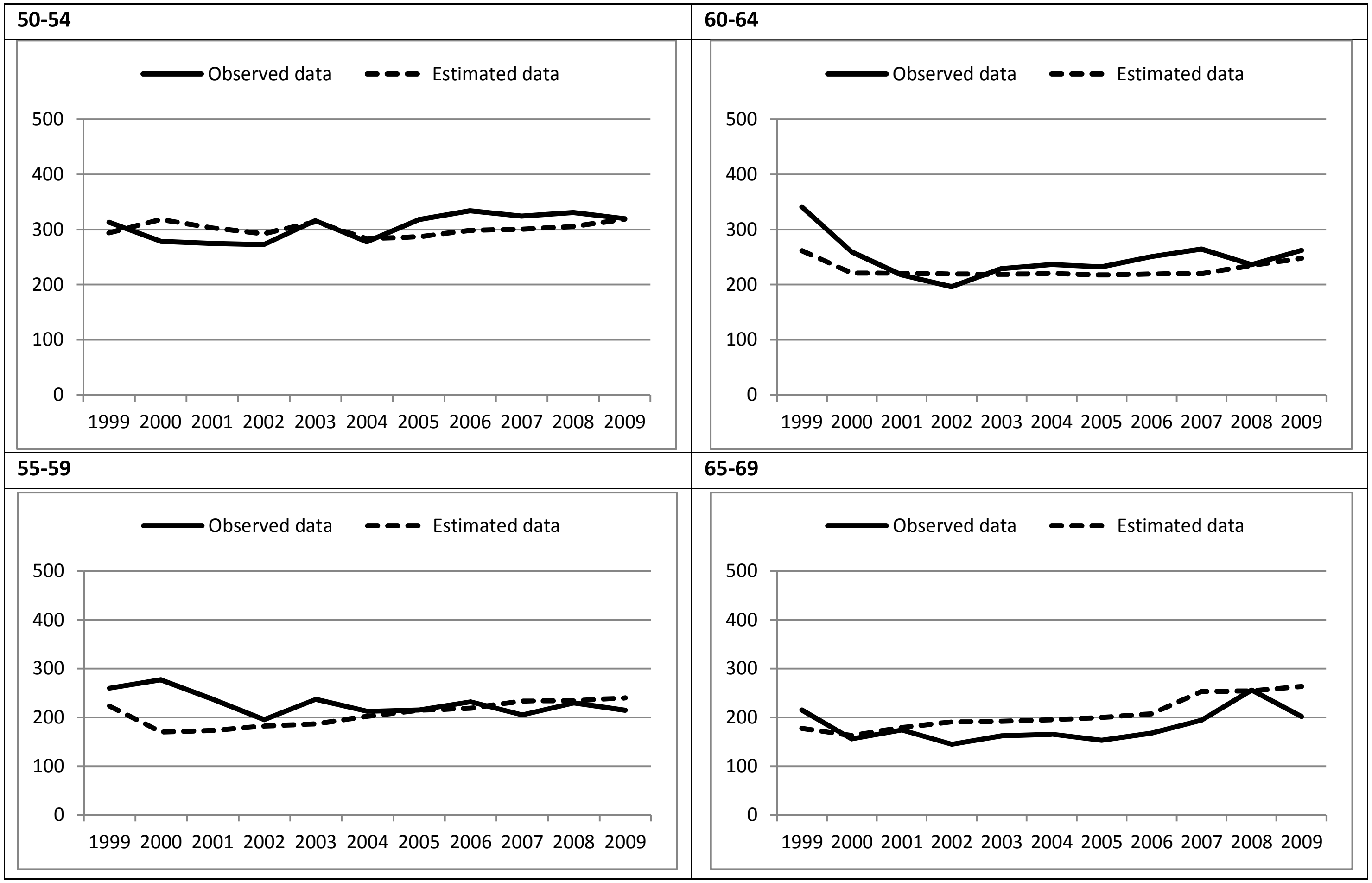

Figure S3: Total number of positive mammogram results in the breast cancer screening programme.

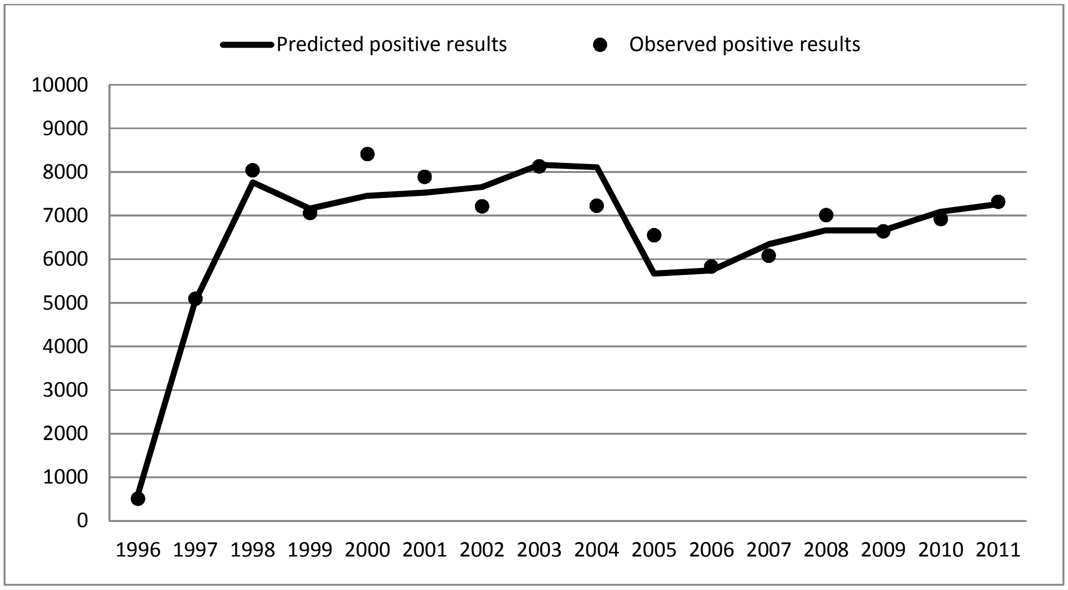

Supplement: Additional file 1: — Model inputs, calibration and validation. This file includes model building details related to the simulation model developed for the evaluation of the Breast Cancer Screening Programme in the Basque Country. In particular, programme specific data used as inputs in the model, a detailed description of the model calibration procedure and goodness-of-fit of the final model are shown in this appendix. (PDF 88 kb) [file 12885_2015_1700_MOESM1_ESM.pdf]
